# Post-Meningitic Syndrome: Pathophysiology and Consequences of Streptococcal Infections on the Central Nervous System

**DOI:** 10.3390/ijms252011053

**Published:** 2024-10-15

**Authors:** Rachid Kaddoura, Karim Abdalbari, Mhmod Kadom, Beshr Abdulaziz Badla, Amin Abu Hijleh, Mohamed Hanifa, Masa AlAshkar, Mohamed Asbaita, Deema Othman, Hanan Faraji, Orjwan AlBakri, Sara Tahlak, Amir Abu Hijleh, Raneem Kabbani, Murtadha Resen, Helmi Abdalbari, Stefan S. Du Plessis, Temidayo S. Omolaoye

**Affiliations:** 1 College of Medicine, Mohammed Bin Rashid University of Medicine and Health Sciences, Dubai Health, Dubai P.O. Box 505055, United Arab Emirates; karim.abdalbari@students.mbru.ac.ae (K.A.); beshr.badla@students.mbru.ac.ae (B.A.B.); amin.abuhejleh@students.mbru.ac.ae (A.A.H.); mohamed.hanifa@students.mbru.ac.ae (M.H.); masa.alashkar@students.mbru.ac.ae (M.A.); mohamed.asbaita@students.mbru.ac.ae (M.A.); deema.othman@students.mbru.ac.ae (D.O.); hanan.faraji@students.mbru.ac.ae (H.F.); orjwan.albakri@students.mbru.ac.ae (O.A.); sara.tahlak@students.mbru.ac.ae (S.T.); amir.abuhejleh@students.mbru.ac.ae (A.A.H.); raneem.kabbani@students.mbru.ac.ae (R.K.); murtadha.resen@students.mbru.ac.ae (M.R.); stefan.duplessis@dubaihealth.ae (S.S.D.P.); 2 Faculty of Medicine, Royal College of Surgeons in Ireland, D02 YN77 Dublin, Ireland; mhmodkadom@rcsi.com; 3 Faculty of Medicine, University of Nicosia, P.O. Box 24005, Nicosia 1700, Cyprus; helmibari@gmail.com

**Keywords:** meningitis, central nervous system, multisystem complications, streptococcal infections, neurology, immunology

## Abstract

Streptococcus species represent a significant global cause of meningitis, leading to brain damage through bacterial virulence factors and the host inflammatory response. Upon entering the central nervous system (CNS), excessive inflammation leads to various neurological and psychological complications. This review explores the pathophysiological mechanisms and associated outcomes of streptococcal meningitis, particularly its short- and long-term neurological sequelae. Neurological symptoms, such as cognitive impairment, motor deficits, and sensory loss, are shown to vary in severity, with children being particularly susceptible to lasting complications. Among survivors, hearing loss, cognitive decline, and cranial nerve palsies emerge as the most frequently reported complications. The findings highlight the need for timely intervention, including neurorehabilitation strategies that focus on optimizing recovery and mitigating long-term disabilities. Future recommendations emphasize improving early diagnosis, expanding vaccine access, and personalizing rehabilitation protocols to enhance patient outcomes. As a novel contribution, this review proposes the term “post-meningitic syndrome” to showcase the broad spectrum of CNS complications that persist following streptococcal meningitis, providing a framework for a future clinical and research focus.

## 1. Introduction

Streptococcal meningitis, a severe, life-threatening infection of the meninges (the protective membranes that cover the brain and spinal cord), is caused by various species of streptococci, including *Streptococcus pneumoniae* and *Streptococcus agalactiae* (group B streptococcus; GBS) [1,2,3]. These bacteria invade the bloodstream and reach the central nervous system (CNS) by breaching the blood–brain barrier (BBB). Once in the CNS, they can trigger a severe inflammatory response, which may result in brain damage, neurological complications, or even death [1,2,3].

In 2019, the global burden of meningitis was significant, with an estimated 236,000 fatalities [4]. Streptococcal infections contributed substantially to this toll. *Streptococcus pneumoniae* accounted for the highest proportion of mortality in all age groups at 18.1% and was the highest in children younger than five years old, accounting for 17.3% of mortalities [4]. Additionally, GBS was the leading cause of neonatal meningitis mortality with 22.8% [4].

The neurological and psychosocial complications of streptococcal meningitis present significant diagnostic and therapeutic challenges due to the variability in symptom presentation [1,3,5]. When assessing the severity and nature of these complications, it is essential to consider both short-term and long-term neurological outcomes, while also considering factors such as age, risk factors, and pathogen type [1,3,5]. The heterogeneity of these symptoms and factors combined with a lack of standardized guidelines for long-term follow-up further hinder early detection and appropriate intervention [3,5,6]. Complications, such as cognitive impairments and behavioral disorders, often manifest subtly and may go unnoticed without consistent monitoring [3,5].

The current literature suggests that multisystem long-term consequences are associated with streptococcal meningitis [1,3,6,7]. For instance, Mohanty et al. conducted a nationwide retrospective cohort study in Sweden, using registry data from 36,230 participants. These included individuals diagnosed with bacterial meningitis before the age of 18 years and general population controls matched at 1:9 by age, sex, and place of residence [7]. Their findings suggest that those diagnosed with bacterial meningitis, especially streptococcal meningitis, are at higher risk of long-term disabilities, including cognitive disabilities, seizures, hearing loss, motor function disorders, visual disturbances, behavioral and emotional disorders, and intracranial structural injuries [7].

The current review aims to provide a comprehensive overview of the epidemiological prevalence of neuropsychological and neurological sequelae in meningitis caused by streptococcal infections. The CNS complications and neuropsychological manifestations following streptococcal meningitis would be better understood by exploring the key factors linking the pathophysiology of meningitis to neurological outcomes. Ultimately, this review seeks to introduce the concept of “Post-meningitic Syndrome”, with the goal of raising awareness about the full spectrum of CNS complications that can arise after streptococcal meningitis.

## 2. Pathophysiology of Streptococcal Meningitis

### 2.1. A Brief Overview of Streptococcal Infections

Streptococcal infections encompass a diverse array of Gram-positive bacteria characterized by their round shape; they are primarily categorized by their cell wall surface antigens [8,9]. Key species include *Streptococcus pneumoniae*, *Streptococcus pyogenes*, and *Streptococcus agalactiae*, all of which are important human pathogens linked to a wide range of illnesses, from mild throat infections to serious conditions such as pneumonia, bacteremia, meningitis, and streptococcal toxic shock syndrome [8,10,11,12]. *S. pneumoniae* is a major cause of community-acquired pneumonia and other severe respiratory issues, while *S. pyogenes* is responsible for over 700 million infections each year, leading to approximately 500,000 deaths due to its virulence and associated public health challenges [10]. *S. agalactiae* primarily affects infants, potentially causing severe conditions like meningitis and sepsis, and poses risks to the elderly and immunocompromised individuals [8,11,13]. Additionally, viridans group members, such as *Streptococcus mutans*, contribute to dental cavities by producing acid from sugar breakdown, illustrating the broad impact of the streptococcal species on human health across various domains [8,14]. 

*S. pyogenes*, also known as Group A Streptococcus (GAS), is a Gram-positive pathogen responsible for a spectrum of human diseases that are historically prevalent in impoverished settings, such as skin infections and rheumatic fever [15]. Although these diseases have declined in developed countries due to improved living conditions and antibiotics, the emergence of new, more virulent strains since the 1980s has sparked global outbreaks of invasive diseases, like necrotizing fasciitis and scarlet fever [15]. *S. agalactiae*, initially identified in cows with mastitis, colonizes the human vaginal tract and has increasingly become pathogenic, particularly in neonates and adults [16]. Both GAS and GBS colonize various mucosal and skin surfaces, with transmission occurring via respiratory droplets and direct contact and potentially through food or insects [17,18]. GAS infections such as pharyngitis spread via oral secretions, while skin infections like cellulitis result from direct invasion through wounds [16]. GBS primarily infects neonates during childbirth but can also cause invasive disease in immunocompromised individuals [19,20]. Key characteristics include GAS’s beta-hemolytic, catalase-negative properties and GBS’s Lancefield classification based on capsular polysaccharides [16,19]. GAS employs virulence factors like the M protein to evade immune responses and induce toxic shock, whereas GBS utilizes capsular polysaccharides and surface proteins for colonization and immune modulation [21,22].

### 2.2. Group A Streptococci

Streptococci, particularly GAS, are responsible for causing a spectrum of symptoms in both adults and children, contributing to an alarming number of annual deaths in the United States, estimated as being between 9000 and 12,000 [23].

GAS, characterized by a wide range of subtypes based on the M-protein, is associated with various conditions, such as acute pharyngitis, impetigo, erysipelas, and cellulitis, along with more severe complications, like streptococcal toxic shock syndrome and necrotizing fasciitis [16]. Additionally, GBS, or *Streptococcus agalactiae*, poses a significant risk for newborn sepsis and postpartum infections [24]. Meanwhile, *Streptococcus pneumoniae*, also known as pneumococcus, remains a primary causative agent of bacterial pneumonia, often linked to otitis media, bacteremia, and meningitis [24]. Among the oral Streptococci, *Streptococcus mutans* plays a pivotal role in dental caries [24].

Furthermore, Enterococcus, which is typically part of normal bowel flora, is increasingly recognized as an opportunistic pathogen, particularly in nosocomial infections [25]. Ongoing efforts to develop effective vaccines highlight Streptococci’s critical role in human health [26]. Regarding the streptococcal strain and meningitis, a diverse array of bacteria within the Streptococcus genus is capable of causing this condition [24]. *Streptococcus pneumoniae* is the most common strain in both adults and children that leads to meningitis worldwide [26]. 

### 2.3. Group B Streptococci

In neonates and young children, GBS, which is a B-hemolytic bacteria, takes the lead as the causative agent of meningitis [24]. Other Streptococcus strains, such as the *Streptococcus viridians* group or GAS, have rarely been observed to cause meningitis across any age group [24].

## 3. Mechanism of Streptococcus Species CNS Invasion and BBB Involvement

The infiltration of the CNS by streptococcal infections involves a complex sequence of events. Initially, mucosal colonization takes place, which leads to systemic dissemination via the bloodstream and the subsequent evasion of CNS barriers facilitated by intricate interactions with host receptors. The CNS, protected by defenses like the BBB and the blood–cerebrospinal fluid barrier, presents a great challenge for invading pathogens [24,27].

Under normal physiological conditions, the brain is considered an immune-privileged site; however, if threatened by meningitis-causing bacteria, an immune response can occur [28]. The BBB serves as a protective shield, separating the brain from the bloodstream to maintain a controlled microenvironment crucial for normal brain function. It is primarily composed of brain microvascular endothelial cells, featuring tight junctions, minimal fenestrae, low pinocytosis, and scant leukocyte adhesion molecules (forming the zona occludens), which effectively partition the bloodstream from cerebral fluid and parenchyma. This separation is further reinforced by the cerebrovascular endothelium, which, in association with human brain vascular pericytes (HBVP), blankets 70% of the abluminal endothelial surface, while astrocyte end-feet furnish an additional shield [29,30]. Additionally, the BBB and the blood–cerebrospinal fluid barriers located at the choroid plexus distinguish blood from CSF using fenestrated endothelial cells. Tight junctions within ventricular epithelial cells restrict cellular and molecular transit to CSF. These tight junctions are composed of three proteins: claudin, occludins, and junction adhesion molecules [31]. Among these proteins, claudins-1, -3, -5, and -12 and occludin are the most critical elements as they are directly involved in regulating the permeability of the tight junction [32]. The brain’s periphery, which is protected by astrocytes, meninges, and CSF spaces, adds another layer of defense along with the non-fenestrated endothelium [29].

Bacteria can cross the BBB by one of three routes: transcellular, paracellular, or through transmigration within infected phagocytes [33,34]. Streptococcus species in particular invade the BBB by migrating transcellularly through a two-step process that involves adherence to the endothelial cells and then lysis by cytotoxic factors [35]. The bacteria are equipped with factors that promote these processes, such as pili or fibrils for adherence and hemolysin or pneumolysin to promote cytotoxicity. This cytotoxic effect leads to dysfunction of the BBB, which affects the membrane integrity, leading to more bacterial invasion [35]. This invasion is detected by pattern recognition receptors (PRR) on the microglia, which leads to their activation. Activated microglial cells then release monocyte chemoattractant protein-1 (MCP-1), tumor necrosis factor (TNF)-α, and interleukin (IL)-6. These cytokines increase the permeability of the microvascular endothelium by reducing claudin-5 and by increasing the expression of adhesion molecules, namely ICAM-1 and VCAM-1 [31,36]. Consequently, more immune cells are recruited, disturbing homeostasis and causing damage to the brain [31].

**Figure 1:**
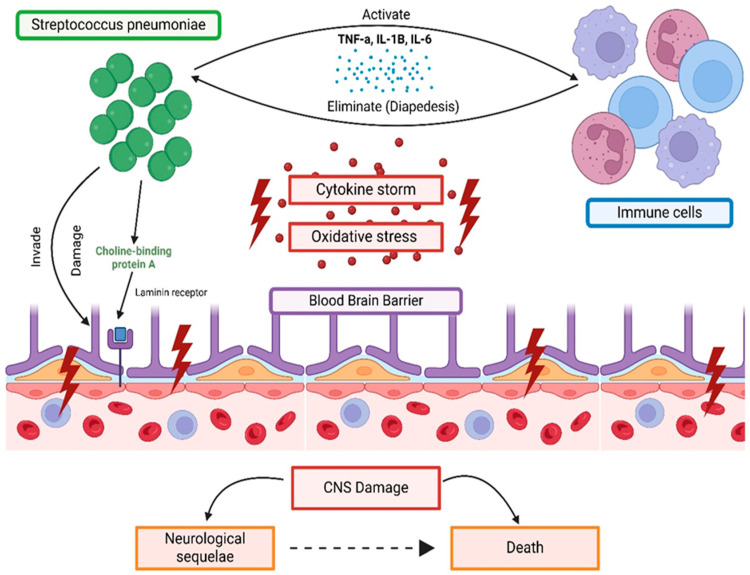
Mechanism of CNS invasion by streptococcus species and BBB involvement. The figure illustrates the mechanism by which *Streptococcus pneumoniae* invades the BBB, causing damage that initially triggers a cytokine storm and increases oxidative stress. This sequence of events eventually leads to CNS damage, which can result in long-term CNS sequelae and potentially death. On the other side of the figure, the role of the immune system in combating the infection is depicted, showing how the bacteria activate the immune response, enabling host cells to eliminate the infection.

The disruption of the BBB is further exacerbated by the activated microglia-producing matrix metalloproteases and reactive oxygen species which lead to its destruction [37]. The consequences of BBB disruption can be severe and can lead to cerebral edema and seizures [24]. Once within the CNS, pathogens exploit the compromised host defense mechanisms, enabling bacterial proliferation and dissemination. Notably, the ability of Streptococci to breach CNS barriers is influenced by factors such as bacteremia levels, age-related alterations, and concurrent systemic inflammation [24,38]. These dynamics underscore the intricate interplay between pathogens and the host’s defenses during CNS invasion. Figure 1 provides a summary of the intricate interaction between CNS invasion by streptococcus species and the BBB.

### Immune Response to Streptococcal Meningitis

Streptococcal meningitis capitalizes on compromised immune defenses in the subarachnoid space, hindering effective complement transfer due to a shortage of soluble PRRs [29]. Despite the increased permeability of the BBB, CSF exhibits reduced complement levels, enabling rapid pathogen proliferation [39]. Immunocompetent cells, equipped with PRRs actively detect pathogens. Toll-like receptors and the nucleotide-binding oligomerization domain (NLRs) sense *Streptococcus pneumoniae*, activating NF-κB and the complement cascade [40]. Elevated concentrations of C5a and C5b-9 correlate with the severity of streptococcal meningitis [41].

The presence of *Streptococcus pneumoniae* triggers a robust inflammatory response in the CNS, marked by an influx of neutrophils, potentially culminating in meningitis syndrome. While neutrophils are critical for defense [29], their role in the pathogenesis and specific recruitment routes to the CNS in meningitis remain specifically unknown. The translocation of neutrophils across the BBB and/or the blood–cerebrospinal fluid barrier (BCSFB) involves intricate steps in the leukocyte adhesion cascade. The challenges posed by the BBB include reduced expression of adhesion molecules and tight junctions in brain microvascular endothelial cells, whereas the BCSFB lacks tight junctions but incorporates barriers from choroid plexus cells. Neutrophils must navigate pericyte layers, the basement membrane, and perivascular space, encountering dynamic challenges. Perivascular cells, including perivascular macrophages and human brain vascular pericytes, actively contribute to CNS inflammation during meningitis [29].

**Figure 2:**
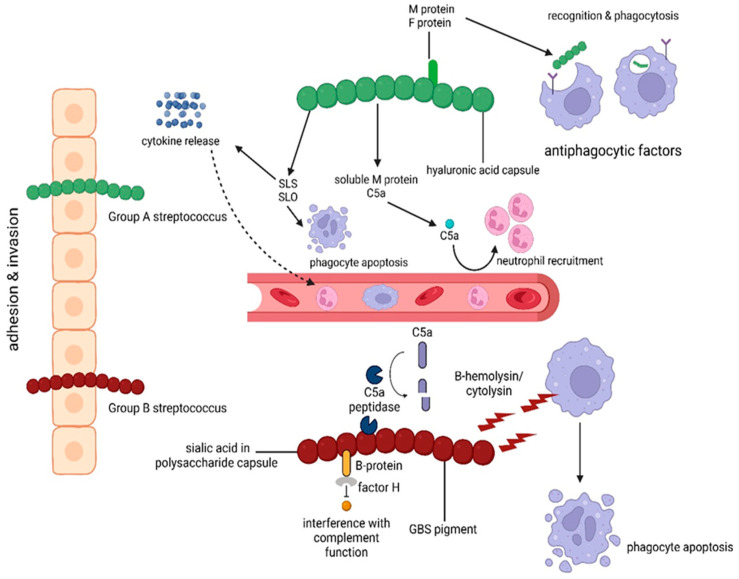
Invasion and immune response to GAS and GBS.

*Streptococcus pneumoniae* emerges as a significant contributor to bacterial meningitis, associated with high mortality and neurological complications. The immediate consequences involve brain edema and septic shock, with subsequent stages potentially leading to cardiopulmonary failure or severe neurological damage. Neuronal injury results from both direct neurotoxicity and the inflammatory response. While microglia, neutrophils, and monocytes are involved, the exact contributions of lymphocytes remain unknown [42]. Figure 2 illustrates the pathophysiology and molecular interaction in the invasion of GAS and GBS.

## 4. Epidemiological Evidence Showcasing Manifestations of Multisystem Dysfunction

Streptococcus meningitis complications tend to affect various organ systems, and many studies have investigated the systemic consequences associated with the condition. The most common of these are cerebrovascular complications [43]. A study examining the incidence of ischemic strokes following streptococcal meningitis infection found that approximately 30% of patients diagnosed with streptococcal meningitis suffered arterial infarctions, 9% experienced cerebral venous thrombosis, and another 9% had cerebral hemorrhages [44]. Although the precise mechanisms are not fully understood, acute phase inflammation likely upregulates the coagulation cascade, leading to a highly thrombotic state and resulting in cerebral infarctions. Endothelin, a vasoconstrictive peptide released by endothelial cells, has been shown to increase the CSF of patients with bacterial meningitis. Additionally, PAI-1, a protein that inhibits tissue plasminogen activator (t-PA), is elevated in the CSF of these patients. This inhibition decreases plasmin levels, heightening the risk of thrombotic events and possibly explaining the increased thrombosis risk [44].

A retrospective study by Pfister et al. investigated the incidence of systemic complications in 87 adult patients diagnosed with pneumococcal meningitis. Thirty-three (37.9%) of the patients experienced meningitis-associated systemic complications and twenty-seven (31%) of those patients were diagnosed with septic shock, highlighting it as one of the most severe complications of the infection [44,45,46]. In severe cases of pneumococcal meningitis, the immune system struggles to contain the bacteria within the CNS. This in turn leads to an overwhelming systemic dissemination resulting in an inflammatory cascade aimed at decreasing bacterial products in the bloodstream. Pathogen-associated molecular patterns (PAMPS) present in bacteria are usually triggers for this cascade. PAMPS bind to Toll-like receptors found on inflammatory cells, leading to the upregulation of pro-inflammatory cytokines, such as IL-1, IL-6, TNF-a, IL-8, and IFN-gamma. The resultant immune response causes a surge in both pro-inflammatory and anti-inflammatory cytokines, leading to multi-organ dysfunction and septic shock. This occurs through the activation of endothelial cells, increased vascular permeability, and leakage of vascular contents into surrounding tissues [47]. Furthermore, 23% of the patients suffered from disseminated intravascular coagulation (DIC). The previously damaged endothelial walls in septic patients can lead to the release of tissue factors into the circulation. As a result, the coagulation pathway is upregulated, leading to further clot formation. About 11.5% of the patients experienced kidney failure and required hemodilution. This could be due to multi-organ failure occurring as a result of sepsis or due to direct bacterial invasion of the kidneys, leading to complications such as pyelonephritis and consequent kidney failure [46]. Lastly, six of the patients developed adult respiratory distress syndrome (ARDS). In a similar manner to the kidney damage, patients with sepsis can develop ARDS due to systemic inflammation, which could in theory affect the lungs, leading to vascular damage and increased permeability, causing fluid accumulation. Another possibility is bacterial invasion, as pneumococcal organisms can use multiple virulent factors and toxins that cause direct damage to lung tissue or the endothelial lining of blood vessels, leading to ARDS.

Cardiovascular complications are also significant after meningitis infections. Studies have indicated that endocarditis, associated with high rates of unfavorable outcomes, such as respiratory failure, pneumonia, and neurological complications, is present in about 2% of patients with bacterial meningitis [48]. The average interval between the diagnosis of streptococcal meningitis and bacterial endocarditis is approximately 16 days, suggesting that endocarditis is a potential complication. Inadequate bacterial clearance in streptococcal meningitis can lead to bacterial dissemination through a compromised BBB, allowing bacteria to enter the bloodstream and form vegetation on heart valves. In the heart, other complications of streptococcal meningitis include myocarditis, arrhythmias, and heart failure. This is most likely due to pneumococcal organisms causing direct damage to heart tissue and the subsequent cardiotoxicity. Pneumonia bacteria translocate through the BBB; strep pneumonia can invade the heart tissue by binding the bacterial adhesin choline-binding protein A to the laminin receptor on vascular endothelial cells. The use of virulence factors such as pneumolysin and hydrogen peroxide can cause cardiomyocyte cell death. Strep pneumonia can also undergo cell wall-mediated inhibition of myocyte contractility [48,49].

An experimental study performed by Hirose et al. explored the ear-related complications involved in pneumococcal meningitis. The findings revealed that the infection could result in significant ear damage, with 60% of mice infected with *S*. *pneumonia* exhibiting hearing loss. This is attributed to fibrosis and ossification of the scala tympani, scala vestibule, and scala media bones of the inner ear [50].

## 5. Neurological Complications

### 5.1. Heterogeneity of Neurological Symptoms

The severity of symptoms in streptococcal meningitis can vary widely among patients. While the classic diagnostic triad in adults includes fever, nuchal rigidity, and altered mental status, only about 44% of adult patients present with all three symptoms. Nearly all patients, however, exhibit at least two out of four key symptoms (fever, headache, neck stiffness, and altered mental status). Clinical signs, such as headache, vomiting, or fever, generally show sensitivity rates of less than 30%, highlighting the heterogeneity in symptom presentation. Signs of meningeal irritation, such as nuchal rigidity and Kernig’s and Brudzinski’s signs, although specific, have low sensitivity. For instance, Thomas et al. found a specificity of 95% and a sensitivity of 5% for both Kernig’s and Brudzinski’s signs, while nuchal rigidity showed a sensitivity of 30% and a specificity of 68% [51]. Given these diagnostic challenges, it is crucial to be aware of the potential short-term neurological complications that may arise with such an infection.

### 5.2. Short-Term Neurological Complications

#### 5.2.1. Short-Term Complications in Children

In children, the short-term complications of bacterial meningitis vary significantly. Subdural effusions and focal neurological deficits are common, with subdural effusions occurring in 20–39% of cases, particularly in infants under one year old. These effusions are often asymptomatic and resolve spontaneously, but intervention may be required in cases of infection, neurological symptoms, or increased intracranial pressure. Focal neurological deficits, presenting as signs localized to specific CNS areas, occur in 3–14% of cases and are often attributed to ischemic stroke or other intracranial lesions. While these deficits typically improve over time, surgical intervention might be necessary for abscesses or empyema, with recovery generally taking longer if ischemic events are present [52,53].

#### 5.2.2. Short-Term Complications in Adults

Among adult patients with bacterial meningitis, Pfister et al. observed 86 patients who suffered from bacterial meningitis and found that CNS complications were detected in 43 individuals [54]. The reported notable CNS complications include cerebrovascular complications (15.1% of patients), brain swelling (14.0%), hydrocephalus (11.6%), and intracerebral hemorrhage (2.3%). Systemic complications, which are quite common, as mentioned earlier, included septic shock (11.6%), adult respiratory distress syndrome (3.5%), and disseminated intravascular coagulation (8.1%). These phenotypes highlight the differences in complication profiles between children and adults, underscoring the importance of age as a factor in the manifestation and severity of bacterial meningitis complications.

#### 5.2.3. Risk Factors for Complications

Identifying patients with risk factors for developing complications of streptococcal meningitis is crucial, as these factors correlate with a higher likelihood of severe complications, ultimately elevating morbidity and mortality rates. A study by Bor et al. analyzing 389 patients with acute bacterial meningitis revealed six significant risk factors for complications such as hydrocephalus, subdural effusions, epilepsy, and septic shock. These risk factors include being below two years of age, leukocytosis, a cerebrospinal fluid glucose level of less than 45 mg/dL, restlessness, rash, and increased incidence of developing hydrocephalus in patients who did not receive steroid therapy before treatment and were being treated with ampicillin-cefotaxime [55]. Additionally, as a review by Zainel et al. on bacterial meningitis in children identified, a delayed presentation, young age, and *S. pneumoniae* as etiological agents are associated with an increased risk for neurological complications such as seizures, hydrocephalus, hearing loss, and mental retardation [43]. 

### 5.3. Long-Term Neurological Complications

#### 5.3.1. Neurological Sequela: Overview

Long-term neurological complications following post-streptococcal meningitis infections are common [56]. The frequently reported persistent neurological sequelae include focal deficits, cognitive impairments, motor deficits, sensory deficits, and seizures [56,57,58,59]. Among patients who develop long-term sequelae, 11–36% develop focal cerebral deficits, 22–69% experience hearing loss, and 4–31% suffer from seizures [56]. Comparative studies have shown that streptococcal meningitis patients are at a greater risk for developing long-term complications compared to those with meningococcal meningitis [59], hence the bacteria that take precedence as the focus of this review. Kolek et al. and Heckenberg et al. reported that 33% and 12% of survivors of meningococcal meningitis exhibited neurological sequelae, respectively [56,60]. 

##### Cognitive Impairment

Cognitive impairment following streptococcal meningitis arises from a complex interplay of pathological mechanisms. The infection triggers a cascade of events, including the production of toxins, inflammatory cytotoxic substances, and brain tissue damage, ultimately leading to neuronal injury and subsequent cognitive decline [59]. In cases of pneumococcal meningitis, the disruption of the BBB initiates CNS inflammation, which increases the expression of receptors for advanced glycation end products in the brain, which, in turn, activates glial cells [61]. The activation of these cells is associated with cognitive changes and impairment [61].

With regard to the cognitive impairment aspect as a neurological complication, similar rates were observed when comparing studies between streptococcal meningitis and meningococcal meningitis. For instance, Lucas et al. reported that the incidence of impairment is 32% in both pneumococcal and meningococcal patient groups [59,62]. Cognitive decline following a bacterial meningitis infection primarily affects mental slowness and memory impairments, regardless of whether the infection is pneumococcal or meningococcal [59,62]. This decline correlates with significant neuronal damage and atrophy of the hippocampus, the brain region crucial for memory and cognitive processes [59,63,64]. 

Kloek et al. found that streptococcal meningitis patients most commonly report impairments in cognitive speed (71%), memory (61%), and attention (60%) [56]. In addition, a study by Van de Beek et al. indicated that cognitive impairment does not necessarily correlate with the overall recovery process [56]. Patients recovering from bacterial meningitis, whether pneumococcal or meningococcal, still exhibited cognitive slowing at similar rates [62].

Cognitive impairment as a neurological sequela in viral meningitis patients is less frequently reported compared to bacterial meningitis. A study by Schmidt et al. found that overall bacterial meningitis exhibited a higher rate of cognitive impairment, particularly in the domains of the learning and memory functions [65]. This could be attributed to multiple different factors, such as the lower severity of viral meningitis, the shorter course of illness in viral meningitis, and the differences in pathophysiology between the two organisms.

#### 5.3.2. Motor and Sensory Deficits

##### Overview of Motor and Sensory Deficits

The motor and sensory sequelae of streptococcal meningitis occur due to multiple processes during acute infection. High levels of inflammation, oxidative stress, and a thrombus-promoting environment with coagulation factors contribute to cerebrovascular infarctions [65]. Pneumococcal-specific cytotoxins, such as pneumolysin, induce neuronal cell death and parenchymal damage, leading to lasting motor and sensory deficits [66].

##### Motor Sequelae in Adult and Pediatric Pneumococcal Meningitis

Motor deficits in adult streptococcal meningitis occur at higher rates compared to those in viral and meningococcal meningitis. Weisfelt et al. reported a 7% incidence of hemiparesis and a 28% incidence of cranial nerve palsies [58]. Cranial nerve palsies can involve both motor and sensory components, but specific palsies are often not detailed in the reports. This means that we should keep this in mind when considering cranial nerve palsies as an indicator of either sensory or motor deficits. Similarly, Jit et al. found high rates of cranial nerve palsies in 12.2% of patients and spasticity/paresis in 8.7% of patients [57]. A follow-up study by Kloek et al. at a median of 2.3 years showed cranial nerve palsies in 29% and hemi/monoparesis in 5%, indicating the long-lasting nature of these motor deficits [56]. 

Streptococcal meningitis is particularly devastating for children, leading to significant long-term morbidity. Stockmann et al. found that 12% of pediatric patients experienced quadriplegia, 12% hemiparesis, 24% cranial nerve palsies, and 8% ataxia following their infection with streptococcal meningitis [67].

##### Meningococcal Meningitis Motor Sequelae in Adults and Pediatrics

Meningococcal meningitis is reported to have more neurological sequelae, and this applies to motor ones too. Edmond et al. reported a 1.8% median risk of motor deficits, and Heckenberg et al. found cranial nerve palsies in 3% of patients’ post-meningococcal meningitis [68,69]. These rates, though significant, are lower than those observed in pneumococcal meningitis, emphasizing the unique impact of different bacterial pathogens on neurological outcomes.

##### Sensory Losses and Pathophysiology of Hearing Loss

Post-streptococcal meningitis can affect all five senses, with hearing being the most frequently affected. Hearing loss following streptococcal meningitis results from bacterial invasion of the cochlea through the cochlear aqueduct, leading to severe suppurative labyrinthitis. This condition damages the sensory hair cells and the neuronal apparatus of the spiral ganglion, causing cell death [52]. Studies involving rat models have reported that reactive oxygen and nitrogen species play a crucial role in inducing this damage to the cochlear apparatus [52].

The rates and severity of hearing loss were investigated by Weisfelt et al., who found that 55% of patients experienced persistent hearing loss at the time of discharge [58]. Follow-up audiometry testing revealed varying degrees of hearing loss severity: mild in 31% of cases, moderate in 21%, severe in 17%, and profound in 31%. Additionally, unilateral hearing abnormalities were noted in 31% of patients [58]. Other studies reported rates of hearing loss of 25% [52], 20.9% [57], and 18.3% [70].

The loss of other senses is less frequently reported than hearing loss. This could be attributed to such sequelae being underrecognized and undertested. A study by Rasmussen et al., which looked at otologic sequelae of streptococcal meningitis, found loss of taste (dysgeusia/ageusia) in 3.9% and loss of smell (dysnosmia/anosmia) in 5.1% of patients [70]. Some patients reported anterior taste loss, potentially implicating the seventh cranial nerve. Vision loss, though infrequently reported, represents a severe complication. Weightman et al. reported a 2.9% rate of blindness; however, in this study, which included children, the age of the patient affected with blindness cannot be ascertained [71]; Edmond et al. reported a 1.7% median risk (1.1–2.1%) for visual disturbances [68].

- Meningococcal meningitis and sensory loss

Meningococcal meningitis also leads to sensory losses but at lower rates than pneumococcal meningitis. Duval et al. found self-reported hearing loss in 15.5% of patients at one-year follow-up [72]. Other studies reported hearing loss rates of 8% [60], a median of 4.6% [68], and 8% [69]. While significant, these rates are lower than that of pneumococcal meningitis, making hearing loss a standout feature of the post-meningitis syndrome of pneumococcal infection. Visual impairment was reported at a median risk of 2.7% [68]. This was slightly higher than pneumococcal infection (1.7%). Cranial nerve palsies were reported by Heckenberg et al. at 3%, which is also an observably lower rate compared to pneumococcal infections [69].

- Pediatric streptococcal meningitis and sensory loss

Sensory sequelae are common and devastating and occur at high rates in children with pneumococcal meningitis, imparting significant morbidity and decreased quality of life. Consistent with the high rates of hearing loss in adults, Stockmann et al. revealed a 29% rate of sensorineural hearing loss in children with 65% of those requiring cochlear implants [67]. They also found a 24% rate of cranial nerve palsies and a 12% rate of cortical blindness [67]. The higher rate of blindness in children highlights this as a unique vulnerability of this age group alongside the other significant sensory losses affecting them.

## 6. Psychosocial and Behavioral Implications

Despite advances in healthcare and the availability of effective antibiotic regimens, the mortality rate of streptococcal meningitis still exceeds 15%. Among survivors, 30–52% experience sequelae; amongst those, psychosocial and behavioral implications are important sequela to keep in mind [73]. Hoogman et al. found that 32% of patients with post-streptococcal meningitis experience cognitive impairment and perform worse on attention/executive function tasks, memory tasks, and psychomotor functions compared to post-meningococcal meningitis patients and healthy controls [62].

Sequalae that are often missed or understated revolve around the psychological impact on the patient. Mental health challenges such as anxiety and depression are prevalent, with Al Janabi et al. reporting that up to 46% of patients with long-term neurological sequelae experience these conditions [74,75]. Another factor to consider is the isolation that occurs, which is in part directly due to the physical limitations and chronic fatigue which can be present independently or in conjunction with the sleep disorders that develop in post-streptococcal meningitis [76,77]. Such isolation is not only physical but also social as patients feel alienated from their friends and family who have not shared their experiences. This isolation and withdrawal has a negative psychological impact on the patient.

**Figure 3:**
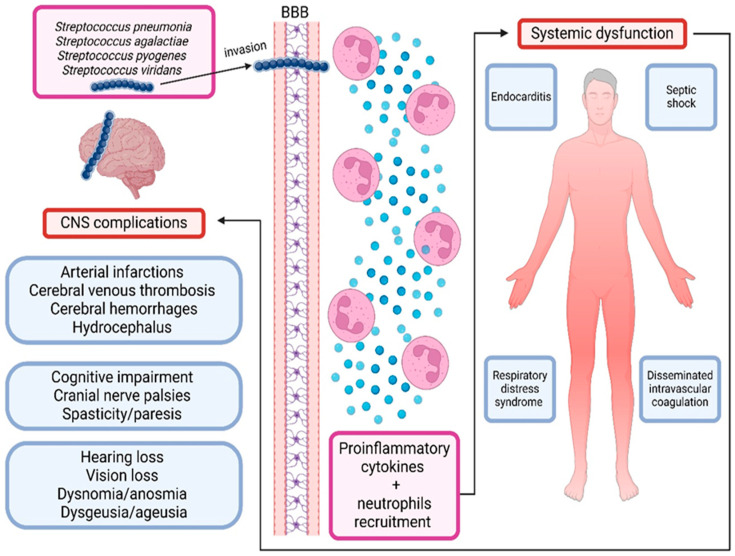
Multisystem complications of streptococcal infections.

Several support programs aim to address such mental health challenges following meningitis. For instance, in the United Kingdom, the Meningitis Research Foundation (MRF) offers counseling programs with trained psychologists and a ‘befriending’ program that connects affected patients with trained MRF befrienders who have had similar experiences. Additionally, staff sometimes provide home visits for face-to-face support [78]. Organizations such as Meningitis Now and the Confederation of Meningitis Organizations provide specialist support tailored to meningitis patients [79]. However, many patients worldwide lack access to such resources and merely rely on individualized coping mechanisms that are not entirely healthy, such as denial, to ease the discomfort of facing reality. Figure 3 provides a summary of the multisystem involvement of streptococcal infections, particularly streptococcal meningitis.

## 7. Diagnostic Challenges and Monitoring: Challenges in Identifying Long-Term Complications

Although early diagnosis and management of streptococcal meningitis tend to improve prognosis and reduce morbidity and mortality, the long-term complications discussed in the current review remain a significant concern. Early detection of these complications is vital to prevent progression. Some challenges hinder the detection of long-term complications, such as inadequate health financing, limited access to diagnostic services, and unequal allocation of healthcare resources, as highlighted by Ocampo et al. [80]. Additionally, a lack of patient awareness of the importance of long-term surveillance of patients with streptococcal meningitis due to poor educational background and an insufficient safety netting is another major challenge observed. This issue could be due to a lack of emphasis within clinical guidelines on the long-term follow-up of these patients. Moreover, complications such as cognitive disabilities and behavioral and emotional disorders are less noticeable and can go undetected for several years [3]. Thus, it is crucial to raise awareness about the need for follow-up care. It is important to note that streptococcal meningitis diagnosis typically relies on a combination of clinical symptoms, CSF analysis, and specific laboratory tests, such as antigen detection, serum or CSF glucose ratio, procalcitonin and C-Reactive protein levels, to identify the causative pathogen. However, when it comes to the various complications discussed in this review, there are currently no validated diagnostic markers, necessitating the importance of future research and further development regarding this matter.

Long-term disabilities in post-streptococcal meningitis can be grouped into six categories, including cognitive disabilities, seizures, hearing loss, motor function disorder, visual disturbance, and behavioral and emotional disorders [80]. In terms of the diagnostic approaches, keeping the mentioned complications in mind and following the traditional path of diagnosing such complications is key. Cognitive disabilities can be diagnosed using the Mini-Cog test which is a three-minute assessment consisting of a recall test and a clock-drawing test [81]. Seizures are typically diagnosed through electroencephalography (EEG) [82]. Hearing loss can be screened using tuning fork tests or audiometry [83]. Motor function disorders can be identified using DSM-5 criteria [84], while visual disturbances require referral to an ophthalmologist for a comprehensive evaluation. Behavioral and emotional disorders should be assessed by a psychiatrist.

Currently, there are no standard guidelines for the follow-up care of patients with bacterial meningitis. However, based on the complications and diagnostic approaches listed above, developing specific guidelines for screening and monitoring patients for long-term complications is crucial. Such guidelines would ensure systematic follow-up and timely interventions, ultimately improving patient outcomes.

## 8. Therapeutic Approaches and Interventions

Up to 30% of survivors of bacterial meningitis, particularly in low- and middle-income countries, often experience neurological or neuro-behavioral sequelae. Neurorehabilitation is suitable for patients with focal neurologic sequelae from bacterial meningitis, cerebral abscess, or subdural empyema [6]. Neurorehabilitation focuses on motor learning to aid motor recovery after brain injury, but understanding how brain injury affects learning, how learning interacts with biological recovery, and how to best incorporate learning principles into rehabilitation protocols remains limited [85]. Rehabilitation for bacterial meningitis involves a multidisciplinary approach that addresses physical, cognitive, and emotional challenges. It aims to enhance recovery by preventing complications, encouraging intrinsic recovery, teaching adaptive methods, and facilitating normal patient function, ultimately optimizing the patient’s function within their usual environment [86]. The treatment includes physical therapy, occupational therapy, speech therapy, cognitive rehabilitation, psychological support, functional training, physical therapy for muscle strength and mobility, occupational therapy for daily living skills, and speech therapy for communication and swallowing challenges [87]. Cognitive rehabilitation focuses on memory, attention, and problem-solving skills. Psychological support helps patients cope with emotional issues, while education provides guidance. Physiotherapy management uses neuroplasticity to reorganize the brain and regain lost functions, targeting areas like neck control, speech, oromotor skills, hypotonia, bed mobility, and balance and coordination [88]. Early integrative neurophysiotherapy, particularly in children, has been successful; it uses a goal-oriented approach to stimulate development and improve mobility and coordination [6].

Plasticity is key in rehabilitating individuals’ post-bacterial meningitis, allowing the brain to reorganize and form new connections to compensate for damage. Inflammation from meningitis can impact cognitive and motor functions and is addressed through rehabilitation strategies leveraging neuroplasticity. Targeted exercises, sensory stimulation, and cognitive tasks aid brain rewiring and function recovery. Plasticity-driven rehab optimizes neural pathways, restoring cognitive and motor skills. This process highlights the importance of tailored interventions in recovery and improving quality of life. Physiotherapy initially focuses on patient consciousness and secondary complications for four weeks. Caregivers are advised on proper positioning every two hours. Subsequently, rehabilitation targets specific areas like neck control, speech, oromotor skills, hypotonia, bed mobility, and balance for two months, emphasizing a comprehensive and structured approach [86].

Functional recovery can occur through impairment resolution or compensation, both of which are responsive to training protocols. Animal models show a brief window of heightened plasticity after focal ischemic damage, which, combined with training, leads to significant motor function gains. In humans, most impairment recovery happens within 3 months post-stroke, suggesting that targeting impairment with intense motor learning protocols during this window could yield substantial gains, akin to those of the animal models [85]. Table 1 provides a summary of the different therapeutic approaches, their focus, and the interventions.

## 9. Future Recommendations

The anticipated global rise in bacterial meningitis cases calls for a more proactive approach to address long-term neurological and psychosocial sequelae. These complications not only affect patients but also burden healthcare systems. A focus on developing therapeutic interventions, particularly neuroprotective and anti-inflammatory treatments, is essential to mitigate acute symptoms and prevent long-term damage. Optimizing rehabilitation during the recovery phase, when neuroplasticity is at its peak, could significantly improve motor and cognitive outcomes.

Early diagnosis remains crucial, as delayed detection increases the risk of lasting neurological impairments. Expanding access to pneumococcal vaccination, the only vaccine-preventable form of streptococcal meningitis, should be prioritized, especially in low-resource regions. This aligns with the WHO’s 2030 roadmap to halve vaccine-preventable meningitis cases and reduce related deaths and disabilities by 70% [89].

Survivors, particularly those with severe sequelae, require long-term, multidisciplinary care, including cognitive and motor rehabilitation, alongside psychological support to manage depression and isolation. Expanding mental health services for these patients will be essential to improving their quality of life. Lastly, future research should explore personalized approaches to meningitis care by identifying genetic and environmental factors that influence susceptibility and recovery. Tailoring prevention and treatment strategies based on these insights could significantly reduce the burden of long-term complications.

## 10. Conclusions

Post-meningitic syndrome is a syndrome that encompasses the clinical neurological and/or psychological sequelae that patients experience following meningitis. The healthcare system is faced with the challenge of identifying such clinical syndromes and addressing the multifaceted needs of affected patients. A holistic approach to patient care is essential, encompassing acute management, long-term rehabilitation, and psychosocial support to improve overall outcomes and quality of life.

## Figures and Tables

**Table 1:** Summary of the therapeutic approaches and interventions for post-meningitis syndrome.

Therapeutic Approach	Focus	Interventions
Physical Therapy	Muscle strength and mobility	Exercises targeting muscle recovery, mobility improvement, and prevention of secondary complications. Early integrative neurophysiotherapy is especially successful in children.
Occupational Therapy	Daily living skills	Training patients in self-care activities, fine motor skills, and adaptive techniques to support independence.
Speech Therapy	Communication and swallowing	Exercises for improving speech production, articulation, language comprehension, and swallowing challenges.
Cognitive Rehabilitation	Memory, attention, and problem solving	Cognitive tasks and exercises to restore memory, attention, problem solving, and executive functioning skills.
Psychological Support	Coping with emotional issues	Psychological counseling, emotional support, and education to assist patients in managing stress, anxiety, or depression following neurological complications.
Neuroplasticity-focused Therapy	Brain reorganization and motor recovery	Targeted exercises, sensory stimulation, and cognitive tasks designed to promote brain rewiring and restoration of motor and cognitive skills.
Early Intervention (Children)	Motor and developmental skills	Goal-oriented approaches to improve mobility and coordination in children through early physiotherapy management and stimulation techniques.
